# Cancer stem cells: mitochondria signalling pathway and strategies for therapeutic interventions

**DOI:** 10.1007/s11033-025-10748-0

**Published:** 2025-07-03

**Authors:** Ee Wern Tan, Sachin Kumar Singh, Kamal Dua, Gaurav Gupta, Wai Leng Lee, Rebecca Shin Yee Wong, Kuan Onn Tan, Bey Hing Goh

**Affiliations:** 1 Sunway Biofunctional Molecules Discovery Centre, Faculty of Medical and Life Sciences, Sunway University, No. 5 Jalan Universiti, Petaling Jaya, Selangor Darul Ehsan 47500 Malaysia; 2 Graduate Institute of Cancer Biology and Drug Discovery, College of Medical Science and Technology, Taipei Medical University, Taipei, Taiwan; 3 Faculty of Health, Australian Research Centre in Complementary and Integrative Medicine, University of Technology Sydney, Ultimo, Australia; 4 School of Pharmaceutical Sciences, Lovely Professional University, Jalandhar - Delhi, Grand Trunk Rd, Phagwara, Punjab 144411 India; 5 Discipline of Pharmacy, Graduate School of Health, University of Technology Sydney, Ultimo, NSW 2007 Australia; 6 Woolcock Institute of Medical Research, Macquarie University, Sydney, NSW 2113 Australia; 7 Centre for Research Impact & Outcome, Chitkara College of Pharmacy, Chitkara University, Rajpura, Punjab 140401 India; 8 School of Science, Monash University Malaysia, Jalan Lagoon Selatan, Bandar Sunway, Subang Jaya, Selangor 47500 Malaysia; 9 Department of Medical Education, Sir Jeffrey Cheah Sunway Medical School, Faculty of Medical and Life Sciences, Sunway University, No. 5 Jalan Universiti, Petaling Jaya, Selangor Darul Ehsan 47500 Malaysia; 10 Department of Biomedical Sciences, Sir Jeffrey Cheah Sunway Medical School, Faculty of Medical and Life Sciences, Sunway University, No. 5 Jalan Universiti, Petaling Jaya, Selangor Darul Ehsan 47500 Malaysia

**Keywords:** Cancer stem cells, Mitochondrial dynamics, Metabolic reprogramming, Therapeutic resistance, Mitochondrial-targeted therapy

## Abstract

Cancer stem cells (CSCs) play a critical role in tumor initiation, progression, and resistance to therapy, making them a major hurdle in effective cancer treatment. Unlike bulk cancer cells, CSCs exhibit remarkable adaptability, allowing them to survive under metabolic stress and evade conventional therapies. Mitochondria, as central regulators of cellular metabolism and apoptosis, are integral to CSC function. They facilitate metabolic reprogramming, redox balance, and stress adaptation, thereby enhancing CSC survival, self-renewal, and resistance to treatment. Dysregulated mitochondrial dynamics, including alterations in biogenesis, degradation, and signaling pathways, contribute to CSC maintenance and therapeutic resistance. Furthermore, mitochondrial membrane integrity and oxidative stress regulation determine CSC fate, influencing their ability to withstand chemotherapy and radiotherapy. Recent advances have identified mitochondrial-targeted strategies as promising approaches to impair CSC function and sensitize them to treatment. These include disrupting mitochondrial metabolism, inducing oxidative stress, and modulating mitochondrial quality control mechanisms. By understanding the intricate relationship between mitochondria and CSCs, new therapeutic strategies can be developed to selectively target CSCs, ultimately improving cancer treatment outcomes and preventing disease recurrence. This review provides an in-depth analysis of mitochondrial mechanisms in CSCs and their potential as therapeutic targets.

## Introduction

Cancer remains one of the leading causes of mortality worldwide, and conventional treatment strategies including radiation therapy, chemotherapy, surgery, and combination therapies continue to form the backbone of cancer management [1, 2]. While these approaches have proven effective in many cases, cancer therapy resistance remains a significant challenge. Despite advances in targeted therapies, chemotherapy remains the most widely used treatment option, yet 90% of chemotherapy failures occur due to drug resistance, particularly during the invasion and metastasis stages of malignancies [1]. This resistance is driven by both genetic and non-genetic mechanisms, which enable cancer cells to evade therapeutic interventions and persist even after aggressive treatments [3, 4].

A major paradigm shift in cancer biology has been the recognition that tumors are not homogenous masses of rapidly dividing cells but rather hierarchically organized heterogeneous populations, with CSCs occupying the apex of this hierarchy [5]. CSCs exhibit self-renewal and differentiation capacities, contributing to tumor initiation, progression, metastasis, and therapy resistance [6]. Unlike bulk tumor cells, CSCs demonstrate enhanced survival mechanisms, allowing them to withstand chemotherapy and radiotherapy, ultimately leading to cancer relapse and recurrence [7].

Among the various factors that contribute to CSC survival and therapeutic resistance, mitochondria play a pivotal role as regulators of cellular metabolism, apoptosis, and stress responses [8]. Mitochondrial function, structure, and dynamics are frequently altered in CSCs, facilitating their metabolic plasticity and adaptation to hostile tumor microenvironments [9, 10]. Furthermore, mitochondria actively regulate crucial pathways, including mitochondrial signaling-induced stress responses, mitophagy-mediated quality control, mitochondrial biogenesis, and metabolic regulatory pathways, which sustain CSC maintenance and therapy resistance. Understanding these mitochondrial mechanisms is critical for developing targeted interventions that selectively eliminate CSCs and enhance cancer treatment efficacy.

This review explores the intricate relationship between CSCs and mitochondrial dynamics, focusing on mitochondrial signaling and stress responses, mitophagy-mediated mitochondrial degradation, mitochondrial biogenesis, and key regulatory pathways that modulate CSC survival and drug resistance. Additionally, we discuss potential therapeutic strategies aimed at disrupting mitochondrial function to enhance CSC eradication and improve cancer treatment outcomes.

## Mitochondrial signaling and the integrated stress response in CSCs

**Fig. 1:**
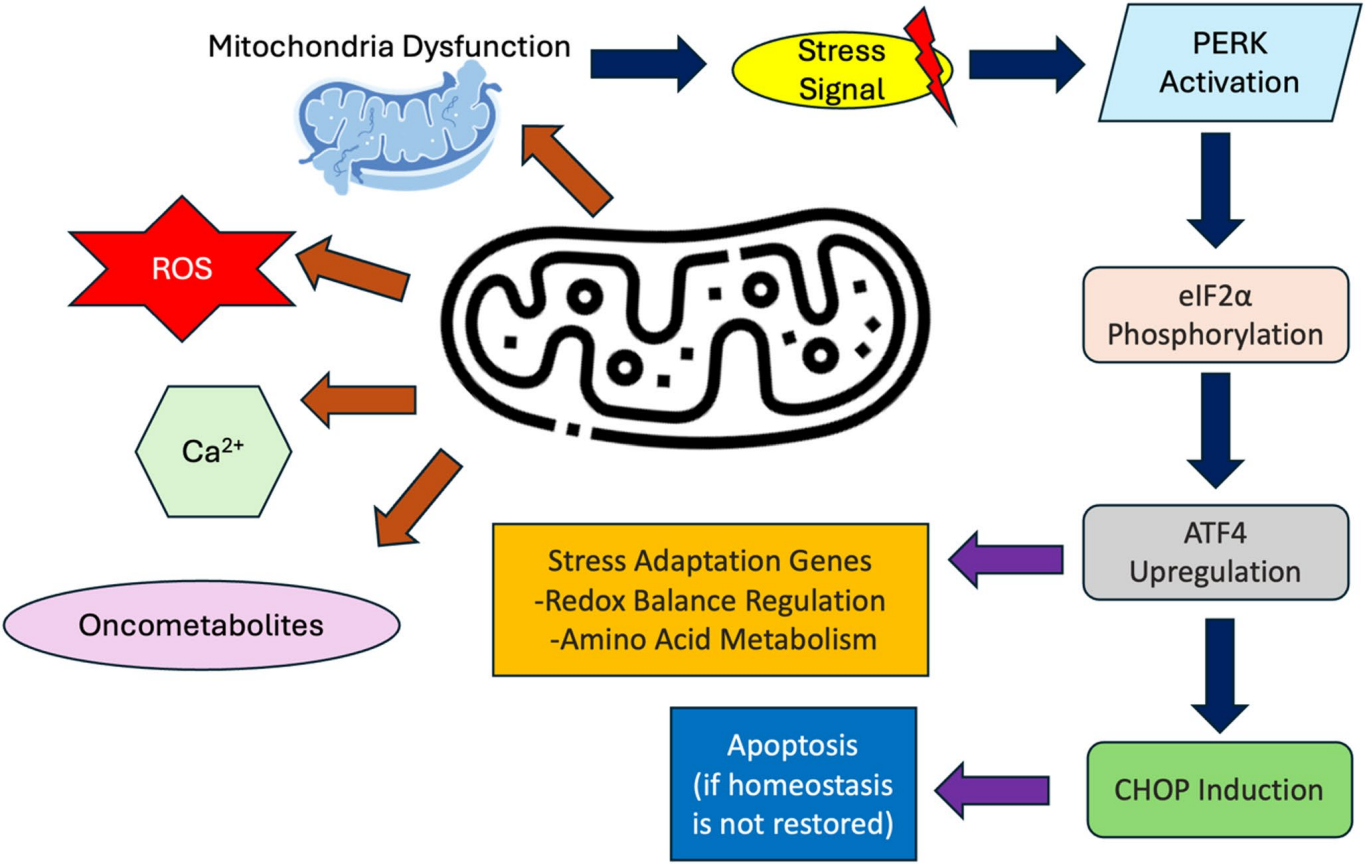
Shows the key components linking mitochondrial signaling to the ISR

Mitochondria serve as essential regulators of cellular metabolism, energy production, and apoptosis. Beyond these primary functions, they also act as critical signaling hubs, particularly in response to cellular stress. One of the key cellular mechanisms influenced by mitochondrial signaling is the Integrated Stress Response (ISR), a conserved pathway that maintains homeostasis under adverse conditions. The ISR is activated in response to various stressors, including mitochondrial dysfunction, oxidative stress, and metabolic disturbances. In cancer, particularly in CSCs, the ISR plays a crucial role in promoting survival, adaptation, and therapy resistance. Understanding the intricate relationship between mitochondrial signaling and the ISR offers new insights into tumor progression and potential therapeutic interventions. The key components linking mitochondrial signaling to the ISR in CSCs is listed in Table 1 and illustrated in Fig. 1.

**Table 1:** Key components linking mitochondrial signaling to the ISR in CSCs

Component	Role in Mitochondrial Signaling and ISR Activation	References
eIF2α Phosphorylation	Reduces global protein synthesis; phosphorylated by kinases like PERK in response to mitochondrial stress.	[11]
ATF4	Transcription factor upregulated during ISR; enhances expression of genes involved in stress adaptation, including those regulating redox balance and amino acid metabolism.	[12, 13]
CHOP	Induced by ATF4 under prolonged stress; promotes apoptosis if cellular homeostasis cannot be restored.	[14]
Reactive Oxygen Species (ROS)	Byproducts of mitochondrial metabolism; elevated levels can damage cellular components, leading to oxidative stress and activation of the ISR.	[15]
Calcium Ions	Mitochondrial dysfunction can disrupt calcium homeostasis, influencing signaling pathways that converge on the ISR.	[16]
Oncometabolites	Accumulation of metabolites like succinate and fumarate can inhibit enzymes involved in maintaining cellular homeostasis, thereby activating stress responses.	[17, 18]

### Mitochondrial dysfunction and ISR activation

Mitochondrial dysfunction can arise from mutations, environmental stressors, or the accumulation of ROS, leading to proteotoxic stress within the mitochondria. This stress results in the activation of the mitochondrial unfolded protein response (UPR^mt^), a protective mechanism that restores mitochondrial function by increasing the expression of chaperone proteins and proteases [19]. However, persistent mitochondrial dysfunction can trigger a broader cellular response, such as the ISR, which regulates global protein synthesis, metabolic pathways, and gene expression to mitigate stress [20]. In cancer cells, including CSCs, the ISR is frequently hijacked to enhance survival and adaptation under unfavorable conditions.

### Key molecular players in mitochondrial-induced ISR

The ISR is primarily regulated by the phosphorylation of eukaryotic initiation factor 2 alpha (eIF2α), which reduces overall protein synthesis to conserve cellular resources [11]. This process is initiated by stress-responsive kinases, such as PKR-like endoplasmic reticulum kinase (PERK), which senses mitochondrial stress signals [21]. As global protein synthesis decreases, specific stress-adaptive proteins, such as activating transcription factor 4 (ATF4), are selectively translated [13]. ATF4 regulates genes involved in redox homeostasis, amino acid metabolism, and apoptosis, helping the cell adapt to metabolic stress [13]. Under prolonged stress conditions, ATF4 induces C/EBP homologous protein (CHOP), a pro-apoptotic factor that determines cell fate by either restoring homeostasis or promoting apoptosis [14].

Mitochondria release various molecules that modulate the ISR. Elevated ROS levels disrupt cellular homeostasis and activate stress-response pathways [19]. Additionally, mitochondrial dysfunction can alter calcium ion (Ca²⁺) homeostasis, which influences ISR signaling [16]. The accumulation of oncometabolites, such as succinate, fumarate, and 2-hydroxyglutarate (2-HG), further disrupts metabolic and epigenetic regulation, leading to the activation of stress-related pathways [18]. These mitochondrial-derived molecules collectively contribute to the activation and maintenance of the ISR in CSCs, enhancing their ability to survive under metabolic stress and resist therapy.

### The ISR in CSCs: adaptation and resistance

CSCs are a subpopulation of tumor cells with self-renewal and differentiation capabilities. They contribute to metastasis, tumor recurrence, and therapy resistance. In CSCs, the ISR plays a dual role, promoting survival and enhancing their ability to withstand metabolic and therapeutic stress [22]. By regulating protein synthesis and stress-adaptive gene expression, the ISR allows CSCs to thrive in nutrient-deprived and hypoxic tumor microenvironments [12]. Moreover, ATF4-driven metabolic adaptation enhances CSC survival by promoting antioxidant defense mechanisms and amino acid biosynthesis [13, 23].

One of the major consequences of ISR activation in CSCs is its role in therapeutic resistance. Conventional treatments, such as chemotherapy and radiation, rely on inducing stress to kill cancer cells. However, CSCs can exploit the ISR to reduce protein synthesis and metabolic activity, making them less susceptible to these treatments [22]. Furthermore, increased mitochondrial biogenesis and the upregulation of mitochondrial antioxidant enzymes allow CSCs to manage oxidative stress more effectively, further enhancing their resistance to therapy [24, 25].

### Therapeutic implications: targeting the ISR in CSCs

Given its role in CSC survival and therapy resistance, targeting the ISR represents a promising strategy for cancer treatment. One approach is to inhibit ISR components, such as blocking eIF2α phosphorylation or disrupting ATF4 activity, which may render CSCs more vulnerable to treatment [11]. For instance, ISR inhibition have been effective in KRAS-driven lung cancer models, reducing tumor growth and improving survival [12]. Besides, combining ISR inhibitors with other treatments, such as chemotherapeutics or immune checkpoint inhibitors, can enhance anti-tumor responses in head and neck squamous cell carcinoma [12]. In addition, blocking ATF4 induction can reduce the cellular response to stressors, as seen with the use of siRNA to inhibit ATF4, which decreased the response to stress-inducing agents like NXP800 in cancer cells [13].

Another strategy is to modulate mitochondrial function by restoring normal oxidative phosphorylation (OXPHOS) or preventing the accumulation of ISR-inducing mitochondrial stress signals [18]. Additionally, targeting oncometabolites that fuel CSC metabolic reprogramming could be a potential therapeutic intervention [18]. The ability of CSCs to withstand metabolic stress and therapy-induced damage is largely driven by mitochondrial quality control mechanisms. The ISR enables CSCs to adapt to stress by modulating protein synthesis and activating survival pathways. However, to sustain long-term survival, CSCs must also regulate mitochondrial homeostasis through mitophagy, ensuring the removal of damaged mitochondria while preserving metabolic efficiency [26]. The synergy between ISR and mitophagy highlights the resilience of CSCs and their ability to evade apoptosis, resist treatment, and maintain their aggressive phenotype.

Given the critical role of mitophagy in CSC survival, disrupting mitochondrial degradation pathways presents a promising therapeutic strategy [26]. Targeting mitophagy could prevent CSCs from clearing dysfunctional mitochondria, leading to metabolic stress and apoptosis [24]. Additionally, combining autophagy inhibitors with conventional therapies could enhance CSC sensitivity to chemotherapy and radiation, thereby reducing tumor recurrence and improving patient outcomes [27, 28]. As research continues, a deeper understanding of mitochondrial dynamics in CSCs will pave the way for novel mitochondria-targeted cancer therapies aimed at eradicating therapy-resistant tumor cells.

## Mitochondrial degradation (mitophagy) in CSCs

**Fig. 2:**
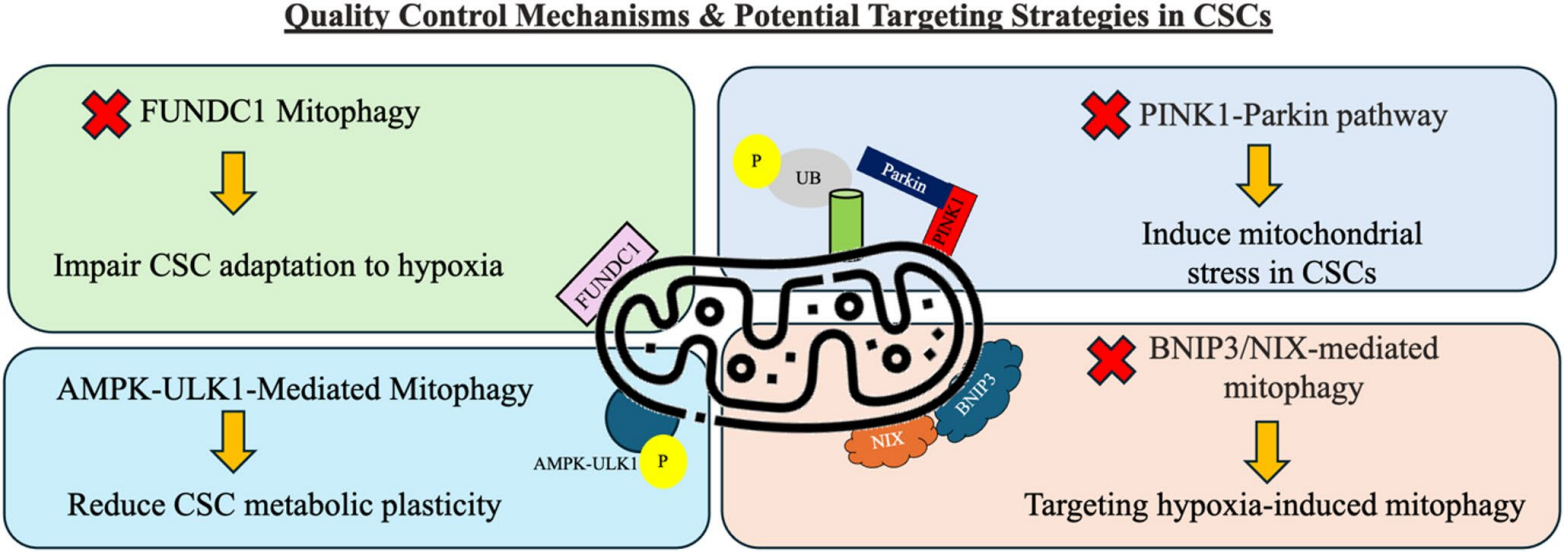
Shows the quality control mechanisms and potential targeting strategies in CSCs

Mitochondrial homeostasis is essential for cellular survival and function, particularly in highly adaptable cell populations like CSCs [26]. Among the various quality control mechanisms that maintain mitochondrial integrity, mitophagy plays a pivotal role in selectively degrading damaged or dysfunctional mitochondria [29]. This tightly regulated process prevents the accumulation of defective mitochondria, thereby maintaining metabolic efficiency and reducing oxidative stress. In CSCs, mitophagy is not merely a maintenance mechanism; it is a crucial factor that supports their self-renewal, metabolic plasticity, and resistance to therapy [24]. Understanding the role of mitophagy in CSCs can provide new insights into their survival strategies and reveal potential vulnerabilities that can be exploited for therapeutic intervention. The key mitophagy pathways in CSCs and their therapeutic implications are listed in Table 2 and illustrated in Fig. 2.

**Table 2:** Key mitophagy pathways in CSCs and their therapeutic implications

Mitophagy Pathway	Key Regulators	Function in CSCs	Therapeutic Implications	References
PINK1-Parkin Mitophagy	PINK1, Parkin	Selectively degrades damaged mitochondria	Inhibiting this pathway can induce mitochondrial stress in CSCs	[26, 30]
BNIP3/NIX Mitophagy	BNIP3, NIX	Active in hypoxia; promotes mitochondrial clearance	Targeting hypoxia-induced mitophagy may enhance CSC elimination	[26]
FUNDC1 Mitophagy	FUNDC1	Hypoxia-inducible mitophagy receptor	Inhibiting FUNDC1 could impair CSC adaptation to hypoxia	[26]
ULK1-Mediated Mitophagy	ULK1, AMPK	Links metabolic stress to mitophagy activation	Targeting AMPK-ULK1 could reduce CSC metabolic plasticity	[26, 31]

### Mitophagy as a quality control mechanism in CSCs

Mitophagy is a selective form of autophagy, a cellular degradation pathway that eliminates damaged organelles and misfolded proteins to maintain homeostasis [29]. This process is particularly vital in CSCs, as their ability to survive under hypoxic conditions, evade apoptosis, and resist therapy relies on efficient mitochondrial turnover [26]. Dysfunctional mitochondria, if not cleared, can lead to excessive ROS production, loss of mitochondrial membrane potential (Δψm), and metabolic collapse [24, 26]. By removing these damaged organelles, mitophagy helps CSCs sustain a functional mitochondrial network, which is essential for their bioenergetic and biosynthetic needs.

The PINK1-Parkin pathway is one of the primary regulatory mechanisms of mitophagy. Under normal conditions, PINK1 is rapidly degraded in healthy mitochondria [32]. However, when mitochondria become damaged, PINK1 accumulates on the outer mitochondrial membrane, recruiting Parkin, an E3 ubiquitin ligase that facilitates the ubiquitination of mitochondrial proteins [30]. This process signals the autophagic machinery to degrade the damaged mitochondria via lysosomes [26]. Studies have shown that CSCs upregulate PINK1-Parkin-dependent mitophagy, allowing them to efficiently eliminate defective mitochondria and sustain their stem-like properties [26, 30].

In addition to the PINK1-Parkin pathway, CSCs also utilize BNIP3/NIX-mediated mitophagy, which is particularly active under hypoxic conditions [26]. BNIP3 and NIX are hypoxia-inducible mitophagy receptors that interact with the autophagic machinery, facilitating mitochondrial clearance in oxygen-deprived environments [26]. Since CSCs thrive in hypoxic tumor niches, this pathway is particularly relevant in their survival and adaptation strategies.

### Mitophagy and CSC metabolic adaptation

CSCs exhibit remarkable metabolic plasticity, allowing them to transition between glycolysis and OXPHOS depending on environmental conditions [26]. Mitophagy plays a crucial role in facilitating this adaptability by selectively removing excess or damaged mitochondria, thereby optimizing energy production and preventing metabolic imbalances [29]. One of the key metabolic benefits of mitophagy in CSCs is its ability to enhance glycolysis. By reducing mitochondrial mass, CSCs can shift toward a predominantly glycolytic metabolism, which is particularly advantageous in hypoxic or nutrient-deprived tumor microenvironments [26]. This metabolic reprogramming enables CSCs to evade mitochondrial-targeting therapies and persist in hostile conditions.

Beyond promoting glycolysis, mitophagy ensures that CSCs maintain a pool of highly functional mitochondria, which is essential for sustaining their bioenergetic and biosynthetic needs. The selective removal of damaged mitochondria preserves mitochondrial efficiency and prevents energy depletion, allowing CSCs to sustain their self-renewal and invasive capabilities [24]. Additionally, mitophagy helps CSCs regulate oxidative stress by eliminating dysfunctional mitochondria that produce excessive ROS [26]. Since an overabundance of ROS can trigger apoptosis, mitophagy serves as a protective mechanism that enhances CSC survival [30]. By tightly regulating mitochondrial quality and metabolic function, mitophagy not only supports the adaptability of CSCs but also contributes to their resistance against metabolic stress and therapeutic interventions.

### Mitophagy in CSC therapy resistance

Mitophagy has emerged as a key mechanism through which CSCs evade apoptosis and resist conventional therapies. Many chemotherapeutic agents and radiation treatments induce mitochondrial damage as a means of killing cancer cells [26]. However, CSCs counteract this by activating mitophagy, thereby removing the damaged mitochondria before they can trigger cell death pathways.

For example, studies have shown that CSCs subjected to mitochondria-targeting drugs, such as OXPHOS inhibitors, exhibit increased mitophagy activity [26]. This compensatory response enables CSCs to survive mitochondrial stress and continue proliferating despite therapeutic intervention. Furthermore, autophagy inhibitors such as chloroquine and hydroxychloroquine have been shown to sensitize CSCs to chemotherapy by blocking mitophagy, thereby enhancing treatment efficacy [27, 28].

### Targeting mitophagy for CSC elimination

Given the crucial role of mitophagy in CSC survival, metabolic adaptation, and therapy resistance, targeting mitophagy represents a promising strategy for CSC elimination. One potential approach is to inhibit key regulators of mitophagy, such as the PINK1-Parkin pathway or BNIP3/NIX-mediated mitophagy [26, 30]. Blocking these pathways could prevent CSCs from effectively clearing damaged mitochondria, leading to mitochondrial dysfunction, excessive ROS accumulation, and increased susceptibility to apoptosis. Additionally, since hypoxia-induced mitophagy is a key survival mechanism for CSCs, disrupting hypoxia-responsive mitophagy regulators like FUNDC1 may further impair CSC adaptation in tumor niches with limited oxygen availability [26].

Beside, exploiting mitophagy-induced vulnerabilities, such as increased reliance on antioxidant defense systems, could offer additional therapeutic opportunities. For example, targeting ROS regulation alongside mitophagy inhibition could push CSCs toward irreversible oxidative damage and apoptosis [30]. In addition, ULK1-mediated mitophagy plays a critical role in maintaining CSC survival by linking metabolic stress to mitochondrial quality control. ULK1, activated by AMP-activated protein kinase (AMPK), facilitates the removal of damaged mitochondria, ensuring efficient energy production and redox balance under stress conditions [26]. This AMPK-ULK1 signaling axis enables CSCs to adapt to fluctuating metabolic demands, enhancing their survival and resistance to therapy [26]. As research continues to uncover the intricate relationship between mitophagy and CSC survival, developing effective mitophagy-targeted therapies could pave the way for improved cancer treatment strategies and reduced tumor recurrence.

## Mitochondrial biogenesis in CSCs

Mitochondrial biogenesis, the process by which new mitochondria are synthesized, is crucial for maintaining cellular energy homeostasis and metabolic adaptability. In CSCs, mitochondrial biogenesis plays a pivotal role in supporting their self-renewal, survival, and resistance to therapy [8]. Unlike differentiated cancer cells, which often exhibit a strong reliance on glycolysis (the Warburg effect), CSCs demonstrate metabolic flexibility by balancing glycolysis and OXPHOS [26]. This adaptability is largely driven by an increased capacity for mitochondrial biogenesis, allowing CSCs to sustain energy demands under varying microenvironmental conditions. Understanding the regulation of mitochondrial biogenesis in CSCs is essential for identifying therapeutic targets to disrupt their metabolic advantages and reduce tumor recurrence. Table 3 shows the key regulators of mitochondrial biogenesis in CSCs and their therapeutic implications.

**Table 3:** Key regulators of mitochondrial biogenesis in CSCs and their therapeutic implications

Regulator	Function in Mitochondrial Biogenesis	Impact on CSCs	Potential Therapeutic Strategy	References
PGC-1α	Master regulator of mitochondrial biogenesis; activates NRF1/NRF2 and TFAM to promote mtDNA replication	Enhances mitochondrial mass and OXPHOS, supporting CSC survival and therapy resistance	PGC-1α inhibitors (e.g., SR18292) to suppress mitochondrial function	[26, 33]
NRF1/NRF2	Stimulates mitochondrial gene transcription and oxidative metabolism	Promotes mitochondrial adaptation to stress, enhancing CSC longevity	NRF inhibitors to disrupt mitochondrial function	[34]
TFAM	Essential for mtDNA maintenance and gene expression	Increases mitochondrial biogenesis and ensures functional mitochondria in CSCs	TFAM-targeting strategies to impair mtDNA replication	[35]
AMPK	Activates PGC-1α to enhance mitochondrial biogenesis in response to metabolic stress	Promotes CSC survival under low-nutrient conditions by increasing mitochondrial efficiency	AMPK inhibition to limit CSC metabolic adaptation	[36]
mTOR	Regulates energy balance and suppresses mitochondrial biogenesis under nutrient-rich conditions	Inhibition of mTOR can induce mitochondrial stress in CSCs, limiting their adaptability	mTOR inhibitors (e.g., rapamycin) to reduce CSC mitochondrial activity	[37]
SIRT1/SIRT3	Regulates mitochondrial metabolism and biogenesis through deacetylation of PGC-1α	Enhances mitochondrial efficiency and protects CSCs from oxidative stress	SIRT inhibitors to sensitize CSCs to therapy	[38]
ERRα	Works with PGC-1α to regulate mitochondrial gene expression	Facilitates mitochondrial adaptation in CSCs	ERRα antagonists to block CSC metabolic flexibility	[39]

### Regulatory pathways governing mitochondrial biogenesis in CSCs

The master regulator of mitochondrial biogenesis is peroxisome proliferator-activated receptor gamma coactivator-1 alpha (PGC-1α). PGC-1α enhances mitochondrial biogenesis by activating nuclear respiratory factors (NRF1 and NRF2), which, in turn, stimulate the transcription of mitochondrial transcription factor A (TFAM), a key regulator of mitochondrial DNA (mtDNA) replication and gene expression [33].

Estrogen-related receptor alpha (ERRα) also interacts with PGC-1α to regulate mitochondrial gene expression, playing a pivotal role in maintaining CSC metabolic plasticity [39]. ERRα enhances mitochondrial adaptation in CSCs, enabling them to efficiently utilize oxidative metabolism and resist metabolic stress [26]. Consequently, targeting ERRα with specific antagonists has been proposed to disrupt CSC metabolic flexibility, potentially impairing their survival and tumor-initiating capacity.

Studies have shown that CSCs often exhibit elevated levels of PGC-1α, leading to an increased mitochondrial mass and enhanced OXPHOS activity [33]. This heightened mitochondrial function supports the energetic and biosynthetic needs of CSCs, enabling them to survive in nutrient-limited tumor microenvironments and resist metabolic stressors.

Apart from PGC-1α, the AMP-activated protein kinase (AMPK) and mechanistic target of rapamycin (mTOR) signaling pathways also contribute to mitochondrial biogenesis in CSCs. AMPK activation, often induced by metabolic stress or low ATP levels, promotes mitochondrial biogenesis by enhancing PGC-1α expression [36]. Conversely, mTOR signaling suppresses mitochondrial biogenesis under nutrient-rich conditions by inhibiting PGC-1α activity [37]. This balance between AMPK and mTOR signaling allows CSCs to fine-tune mitochondrial biogenesis according to environmental cues, optimizing their survival in both hypoxic and nutrient-sufficient conditions.

Additionally, SIRT1 and SIRT3 have emerged as critical regulators of mitochondrial metabolism and biogenesis in CSCs through their deacetylation of PGC-1α, enhancing mitochondrial efficiency and protecting CSCs from oxidative stress [38]. By maintaining mitochondrial function, SIRT1/SIRT3 activity enables CSCs to adapt to metabolic challenges, reinforcing their survival and therapy resistance [40]. Targeting SIRT1/SIRT3 with specific inhibitors has been proposed as a strategy to disrupt CSC mitochondrial resilience, thereby sensitizing them to conventional treatments [38].

### Mitochondrial biogenesis and CSC therapy resistance

One of the defining characteristics of CSCs is their remarkable resistance to conventional cancer therapies, including chemotherapy and radiation. Recent studies have linked this resistance to enhanced mitochondrial biogenesis, which provides CSCs with increased energy production, antioxidant defenses, and apoptotic resistance. For instance, higher mitochondrial biogenesis has been associated with greater mitochondrial spare respiratory capacity, allowing CSCs to rapidly adapt to oxidative stress induced by anticancer treatments [41]. Additionally, CSCs with elevated mitochondrial biogenesis exhibit lower levels of mitochondrial dysfunction after drug exposure, enabling them to evade apoptosis and repopulate the tumor after treatment [24].

Mitochondrial biogenesis also contributes to CSC resistance by modulating ROS levels. While excessive ROS can lead to cellular damage and apoptosis, CSCs utilize mitochondrial biogenesis to maintain ROS homeostasis [26]. By constantly generating new, functional mitochondria and degrading damaged ones through mitophagy, CSCs can regulate ROS levels and avoid oxidative stress-induced cell death. This protective mechanism further enhances their survival and underscores the need for therapeutic strategies targeting mitochondrial biogenesis.

### Targeting mitochondrial biogenesis as a therapeutic strategy

Given the role of mitochondrial biogenesis in CSC survival and therapy resistance, disrupting this process has emerged as a potential strategy for eliminating CSCs. Several approaches have been proposed to target mitochondrial biogenesis, including the inhibition of PGC-1α, NRF1/NRF2, and TFAM [35]. Small-molecule inhibitors, such as SR18292, have been shown to suppress PGC-1α activity, leading to impaired mitochondrial biogenesis and reduced CSC viability [42]. Similarly, blocking NRF1/NRF2 signaling disrupts mtDNA replication and mitochondrial gene expression, impairing the ability of CSCs to sustain their metabolic demands [34].

Another promising strategy involves the use of AMPK and mTOR modulators. Since AMPK activation promotes mitochondrial biogenesis, inhibiting AMPK signaling may suppress CSC mitochondrial function and reduce their metabolic plasticity [36]. On the other hand, mTOR inhibitors, such as rapamycin and its analogs, have been found to decrease mitochondrial biogenesis and sensitize CSCs to conventional therapies [37]. Targeting mitochondrial biogenesis in combination with chemotherapy or radiotherapy could enhance treatment efficacy by preventing CSCs from restoring their mitochondrial network after drug-induced damage.

Additionally, emerging evidence suggests that disrupting mitochondrial dynamics, specifically the balance between biogenesis and mitophagy can effectively impair CSC metabolism [26]. By simultaneously inhibiting mitochondrial biogenesis and enhancing mitophagy, CSCs can be forced into metabolic crisis, leading to energy depletion and cell death. This dual-targeting approach may offer a novel therapeutic avenue for eradicating CSCs and reducing tumor relapse.

## Mitochondrial regulatory pathways in CSCs

Mitochondria play a crucial role in regulating CSC survival, metabolism, and stress responses, enabling them to adapt to harsh microenvironments and resist therapy [43]. Key mitochondrial processes, such as fission and fusion, are tightly regulated by proteins like dynamin-related protein 1 (DRP1), which controls mitochondrial division and mitophagy, ensuring metabolic flexibility in CSCs [44]. Additionally, OXPHOS and ROS generation contribute to CSC maintenance, with antioxidant enzymes like Peroxiredoxin 3 (PRDX3) counteracting oxidative stress [33, 45]. Another key regulator, voltage-dependent anion channel 1 (VDAC1), governs mitochondrial membrane permeability and metabolic exchange, influencing CSC survival and apoptosis resistance [46].

**Fig. 3:**
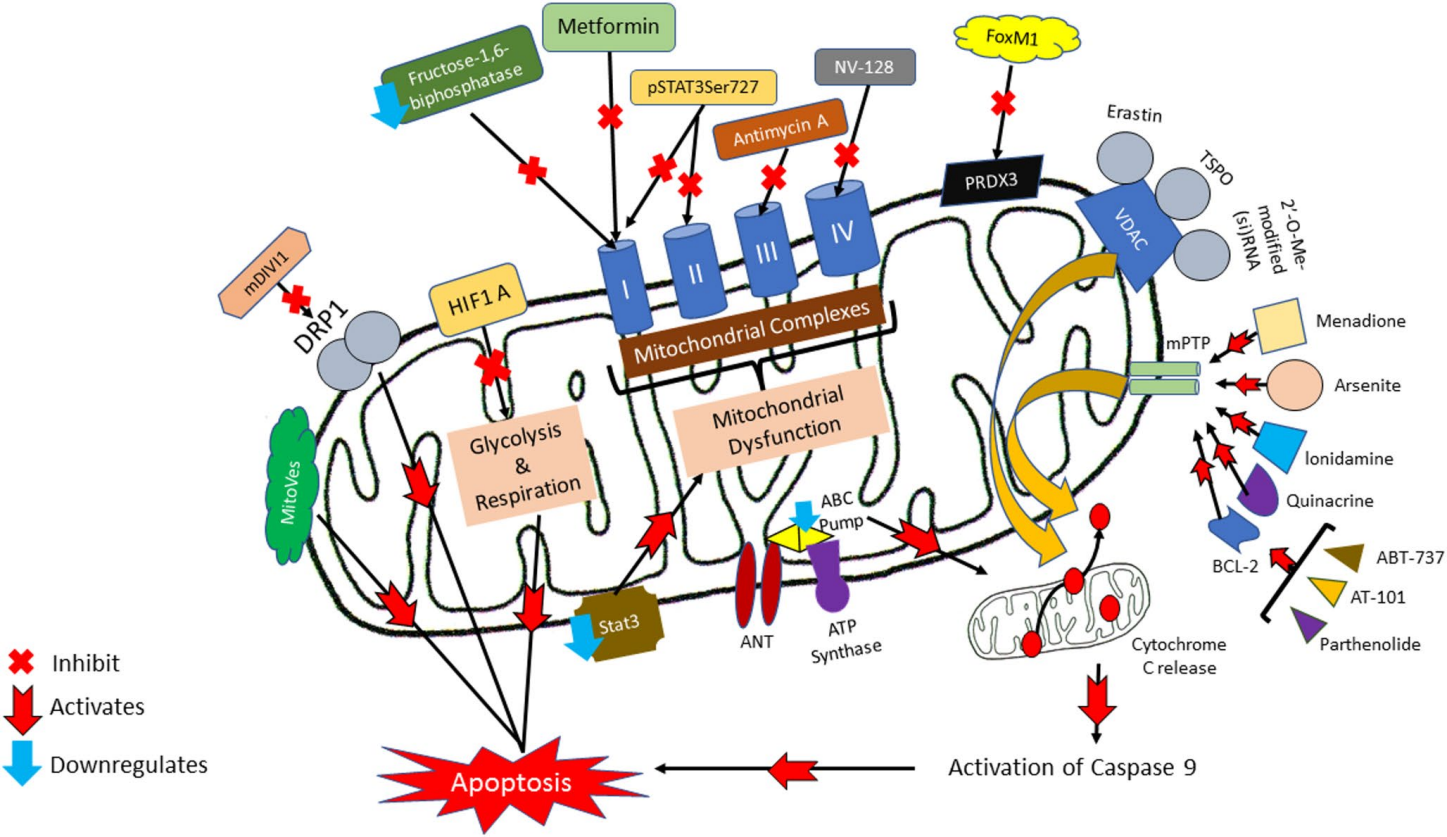
Shows the summary of mitochondria signaling and strategies for therapeutic interventions

Beyond metabolic control, mitochondrial-mediated apoptosis in CSCs is tightly regulated by Bcl-2 family proteins, preventing programmed cell death and contributing to therapy resistance [47]. Mitochondrial membrane permeability transition (MPT) also plays a crucial role in apoptosis regulation [48], while epigenetic modifications further reshape mitochondrial function to sustain CSC properties [49]. Targeting mitochondrial vulnerabilities has emerged as a promising therapeutic strategy, with anti-cancer drugs inducing mitochondrial dysfunction to selectively eliminate CSCs. Additionally, tumor necrosis factor receptor (TNFR) signaling pathways influence CSC survival by modulating mitochondrial responses to inflammation and stress. The following sections will explore these mitochondrial regulatory mechanisms in detail, highlighting their roles in CSC biology and their potential as therapeutic targets. The summary of mitochondria regulatory pathways and the strategies for therapeutic interventions are listed in Table 4 below and illustrated in Fig. 3.

**Table 4:** Summary of mitochondria regulatory pathways and the strategies for therapeutic interventions

Pathways	Pathway’s Description	Strategies/Drugs	Strategy’s Descriptions	Type of Cancers	Reference
Dynamin-related protein 1 (DRP1)	Constriction and fission of mitochondria confer DRP1 chemoresistance	Mitochondrial DIVision inhibitor 1	Inhibits DRP1 activity	Breast, lung colon, liver, prostate, pancreas, ovarian	[44, 50]
OXPHOS & ROS	Essential for CSC growth and maintenance	Targets on HIF-1α	Selectively block glycolysis and mitochondrial respiration	Breast	[51]
		Antimycin A	Inhibit mitochondria complex III		[52]
Peroxiredoxin 3 (PRDX3)	Localized in mitochondria which is the main source of ROS for CSC maintenance	Downregulates forkhead box M1 (FOXM1)	Inhibit transcription that activates PRDX3 and CSC marker, CD 133	Colon	[25, 53]
Voltage-dependent anion channel 1 (VDAC1)	Maintain metabolic and cell energy homeostasis	Erastin	To induce oxidative stress and caspase-9 dependent cell apoptosis	Glioblastoma	[54]
	Highly expressed in tumours due to its high energy requirement specifically for cancer cells	Translocator protein (TSPO)	To regulate cholesterol transport, mitochondrial respiration, and apoptosis	Liver, prostate, kidney and brain	[55]
		2’-O-Me-modified short interfering (si)RNA to reduced in cellular ATP levels and cell growth	VDAC1 depletion leads to mitochondria metabolic reprograming	Lung, glioblastoma	[56, 57]
Mitochondrial membrane permeability transition	Activation of pro-apoptotic proteins in the mitochondria results in the opening of mPTP	Menadione, arsenite and lonidamine	Activation of mPTP subsequently activating apoptotic processes	Glioblastoma	[58–60]
	To induce mitochondrial permeabilization via bioactive substances	ATP synthase and adenine nucleotide translocator (ANT)	Downregulate ATP binding cassette (ABC) pumps	-	[61]
Proteins of the Bcl-2 family	Increased Bak and Bax activity to promote apoptosis	Overexpression of anti-apoptotic proteins from Bcl-2 family	Interact with mPTP core components	Leukemia, breast	[62–64]
		ABT-737 and Gossypol (AT-101)	Interact with BCL-XL and BCL-2	Leukemia	[65]
		Parthenolide	Activates proapoptotic protein Bax	Acute myelogenous leukemia, breast, melanoma initiating-cancer cells, osteosarcoma	[66–68]
		Quinacrine	Modulate Bcl-2 family protein activity	Leukemia, oral, breast	[69–71]
Mitochondrial dysfunction	Internal and external stimuli can promote cell death, reducing electron transport chain efficiency and high-energy molecule production.	Ceramide, CD437 and MKT077	Anti-cancer drugs to induce mitochondria dysfunction	-	[72–74]
		Etoposide, paclitaxel, and vinorelbine	Clinical approved drugs	-	[75]
		Downregulates fructose-1,6-biphosphatase	Induces glycolysis and suppresses mitochondrial Complex I activity	Breast	[76]
		Metformin	AMPK activator and Complex I inhibitor	-	[77]
		Vitamin E succinate (MitoVES)	Toxic agents to induce apoptosis	Breast	[78, 79]
		Isoflavone derivative NV-128	Decreases ATP, Complex I and IV levels	Ovarian	[80]
		Taxoid SB-T-1214	Reduces stemness gene expression	-	[81]
		EpCAM	Reduces proliferation and differentiation of CSCs	Colorectal, ovarian	[82, 83]
Tumour necrosis factor receptor (TNFR)	TNF induces cell death, survival, differentiation, and proliferation in all cell types.	Downregulates TNFR2/STAT3	Altering mitochondrial ultrastructure, translocating cytochrome C to the cytoplasm, activating cleaved caspase 3p175, phosphorylating MLKLSer358, and generating ROS.	Renal	[84]
	TNF promotes cell death and survival via binding to TNFR1 and TNFR2, respectively	De-phosphorylate STAT3 on serine-727 (pSTAT3Ser727)	ETC Complex I and II inhibition	Glioblastoma	[85, 86]

### DRP1: regulator of mitochondrial fussion and fission machinery

Dynamin-related protein 1 (DRP1) is a GTPase that plays a pivotal role in mitochondrial dynamics by mediating mitochondrial fission, a process essential for maintaining mitochondrial function and cellular homeostasis [44]. In CSCs, DRP1-driven mitochondrial fission is crucial for regulating metabolism, proliferation, and survival [44]. The activity of DRP1 is modulated through various post-translational modifications, including phosphorylation, which influence its interaction with signaling pathways that govern CSC maintenance. For instance, phosphorylation of DRP1 at serine 616 has been associated with enhanced mitochondrial fragmentation and increased stemness in cancer cells [87, 88].

Targeting DRP1 has emerged as a potential therapeutic strategy to disrupt mitochondrial dynamics in CSCs. Mitochondrial division inhibitor 1 (mdivi-1), a small molecule inhibitor of DRP1, has been shown to induce mitochondrial elongation and impair mitochondrial fission [89]. Recent studies have demonstrated that mdivi-1 treatment leads to decreased cell viability and increased apoptosis in various cancer cell lines, including breast, lung, colon, pancreatic and ovarian cancers, respectively, while sparing normal cells [44, 90, 91]. These findings suggest that inhibiting DRP1-mediated mitochondrial fission can selectively target CSCs and sensitize them to apoptosis.

Furthermore, DRP1 interacts with multiple signaling pathways that modulate CSC survival. For example, the mitogen-activated protein kinase (MAPK) pathway has been implicated in the phosphorylation of DRP1, leading to mitochondrial fragmentation and the promotion of stemness in cancer cells [92]. Inhibition of DRP1 disrupts these signaling interactions, resulting in reduced mitochondrial fission and decreased CSC viability offering a promising approach to impair mitochondrial dynamics and selectively eliminate CSCs, highlighting the potential of DRP1 as a therapeutic target in cancer treatment [50, 93].

### OXPHOS and ROS in mitochondria

OXPHOS is a primary source of ROS within cells. In CSCs, ROS levels are intricately regulated through specific signaling pathways to maintain a balance that supports tumor progression. Elevated ROS can induce cellular damage and apoptosis, while moderate ROS levels can promote CSC survival, proliferation, and metastasis [29]. One critical regulator in this context is hypoxia-inducible factor 1-alpha (HIF-1α), a transcription factor stabilized under low oxygen conditions. HIF-1α activation leads to the transcription of genes that facilitate adaptation to hypoxia, including those involved in angiogenesis, metabolism, and survival pathways [51]. Notably, ROS can stabilize HIF-1α, creating a feedback loop that enhances CSC properties and contributes to tumor progression [51].

Targeting the OXPHOS pathway to modulate ROS levels has been explored as a therapeutic strategy against CSCs. Antimycin A, an inhibitor of complex III in the electron transport chain, increases ROS production by blocking electron flow, leading to elevated ROS levels that can surpass the threshold tolerable by CSCs, thereby inducing cell death [52]. Recent studies have investigated the effects of Antimycin A on various cancer types. For instance, research published in 2021 demonstrated that Antimycin A treatment in breast cancer cell lines resulted in increased ROS production, leading to decreased cell viability and induction of apoptosis [52]. These findings suggest that manipulating ROS levels through targeted interventions in the OXPHOS pathway can disrupt CSC maintenance and impede tumor progression.

### Peroxiredoxin 3 (PRDX3) for CSCs maintenance

Peroxiredoxin 3 (PRDX3), a mitochondrial antioxidant enzyme, plays a crucial role in regulating ROS levels and maintaining redox homeostasis within CSCs. PRDX3 is highly expressed in CSCs and has been implicated in promoting their survival, proliferation, and therapy resistance. By scavenging excessive mitochondrial ROS, PRDX3 prevents oxidative damage and sustains the metabolic and stem-like properties of CSCs. Studies have demonstrated that PRDX3 expression is particularly elevated in CSC-enriched populations, such as CD133⁺ colon cancer stem cells, where it facilitates tumor progression and metastasis [53]. Additionally, PRDX3 enhances mitochondrial function by stabilizing OXPHOS, ensuring a steady supply of ATP that supports CSC maintenance [9].

One promising approach to targeting PRDX3 in CSCs is the downregulation of Forkhead Box M1 (FOXM1), a transcription factor that regulates PRDX3 expression. FOXM1 is known to enhance CSC traits by promoting self-renewal, drug resistance, and tumor initiation. Recent studies have shown that FOXM1 inhibition leads to a significant reduction in PRDX3 levels, thereby increasing mitochondrial ROS accumulation and triggering oxidative stress-induced apoptosis in CSCs [25]. In a study on colon cancer, suppression of FOXM1 led to reduced PRDX3 expression in CD133⁺ CSCs, resulting in decreased tumor sphere formation and sensitization to chemotherapy [53]. Another study on glioblastoma CSCs found that PRDX3 knockdown inhibited self-renewal and impaired mitochondrial function, further supporting its essential role in CSC maintenance [9].

Beyond FOXM1 inhibition, other strategies have been explored to disrupt PRDX3 function in CSCs. Small-molecule inhibitors targeting PRDX3 have demonstrated potential in preclinical models, leading to increased ROS-induced cell death and reduced tumor burden [9]. Additionally, combining PRDX3 inhibition with standard chemotherapy or radiotherapy has been shown to enhance treatment efficacy by overcoming CSC-associated resistance mechanisms. In a recent study on colorectal cancer, PRDX3 inhibition sensitized CSCs to 5-fluorouracil (5-FU), resulting in a significant reduction in tumor growth [53]. Taken together, future studies focusing on PRDX3-targeting agents, either as monotherapy or in combination with existing treatments, could pave the way for more effective cancer therapies.

### VDAC1

Voltage-dependent anion channel 1 (VDAC1) is a crucial mitochondrial outer membrane protein that regulates metabolic and energetic processes in CSCs. By controlling the exchange of metabolites and ions between the mitochondria and cytosol, VDAC1 plays a fundamental role in maintaining mitochondrial function, ATP production, and cell survival. Given the high energy demands of CSCs, VDAC1 is often overexpressed in various cancers, including glioblastoma, cervical, and lung cancers, where it facilitates tumor progression and therapy resistance [54, 94]. Recent studies have demonstrated that targeting VDAC1 can disrupt mitochondrial metabolism, induce oxidative stress, and trigger apoptosis, making it an attractive therapeutic target for CSC eradication [54].

One of the key strategies to target VDAC1 is through the use of Erastin, a small molecule known to bind and modulate VDAC1 function. Erastin promotes oxidative stress by enhancing ROS production and disrupting mitochondrial permeability, leading to caspase-9-dependent apoptosis in CSCs. In a 2021 study on glioblastoma CSCs, treatment with Erastin significantly reduced cell viability and impaired tumor sphere formation by inducing mitochondrial dysfunction [54]. Similarly, in cervical cancer CSCs, Erastin-mediated VDAC1 modulation resulted in elevated ROS levels, leading to apoptotic cell death and decreased tumor growth in in vivo models [54, 95]. These findings highlight the potential of Erastin as a metabolic disruptor in CSC-targeted therapy.

The translocator protein (TSPO) is another mitochondrial outer membrane protein closely linked to VDAC1 and is highly expressed in tumors due to their elevated energy requirements. TSPO interacts with VDAC1 to regulate cholesterol transport, mitochondrial respiration, and apoptosis. Studies have shown that high TSPO expression is associated with CSC maintenance and chemoresistance, particularly in liver, prostate, kidney and brain cancer [96]. Inhibition of TSPO has been found to reduce CSC proliferation and sensitize tumors to chemotherapy, indicating its potential as a co-target in VDAC1-directed therapies [96].

Another approach to targeting VDAC1 in CSCs involves gene silencing strategies such as 2′-O-Me-modified short interfering (si)RNA, which effectively downregulates VDAC1 expression. VDAC1 depletion has been shown to disrupt mitochondrial metabolic homeostasis, leading to reduced ATP production and impaired CSC growth. In a 2022 study on lung cancer stem-like cells, siRNA-mediated VDAC1 knockdown resulted in metabolic reprogramming, characterized by a shift from OXPHOS to glycolysis, ultimately impairing tumor progression [56]. Similar findings were observed in glioblastoma CSCs, where VDAC1 silencing led to a decrease in mitochondrial ATP production and increased sensitivity to chemotherapy [57, 94].

### Mitochondrial membrane permeability transition

Mitochondrial membrane permeability transition (mPT) is a crucial event in cellular homeostasis and apoptosis, particularly in CSCs, where mitochondrial integrity plays a role in survival, therapy resistance, and metabolic adaptation. The opening of the mitochondrial permeability transition pore (mPTP), a high-conductance channel in the inner mitochondrial membrane, leads to mitochondrial swelling, membrane depolarization, and subsequent cell death. In CSCs, mPTP opening is tightly regulated by pro-apoptotic proteins, metabolic stress, and interactions with oncogenic signaling pathways. Several bioactive compounds, including menadione, arsenite, and lonidamine, have been shown to activate pro-apoptotic pathways and trigger mPTP opening, leading to CSC elimination [58, 59]. For instance, a study by Jane et al. (2023) demonstrated that lonidamine enhances mitochondrial dysfunction and apoptosis in glioblastoma stem-like cells by promoting mPTP opening and increasing ROS levels, thereby sensitizing cells to chemotherapeutic agents [97].

Key mitochondrial components, such as ATP synthase and adenine nucleotide translocator (ANT), also play pivotal roles in mPTP regulation by influencing mitochondrial membrane integrity and bioenergetics. ATP synthase, apart from its role in OXPHOS, has been implicated in mPTP formation, with its c-subunit interacting with cyclophilin D (CypD) to regulate pore opening. Additionally, ANT modulates mitochondrial permeability by facilitating the exchange of ADP and ATP across the inner membrane, and its interaction with CypD further influences mPTP dynamics. Downregulation of ATP-binding cassette (ABC) transporters, a key resistance mechanism in CSCs, has been associated with enhanced mitochondrial permeabilization, leading to cell death [61]. For instance, a study found that arsenite treatment downregulated ABC transporters, induced mitochondrial permeabilization via ANT activation, and triggered apoptosis through caspase-9-dependent pathways [58]. Similarly, menadione was shown to disrupt ATP synthase function, reducing ATP production and driving metabolic collapse [59]. These findings highlight the potential of targeting mPTP dynamics and its regulatory proteins as a therapeutic strategy against CSCs, offering a means to overcome drug resistance and induce selective cancer cell apoptosis.

### Proteins of the Bcl-2 family

The Bcl-2 family of proteins plays a crucial role in the regulation of apoptosis, particularly in CSCs, where their balance between pro-apoptotic and anti-apoptotic members dictates cell survival. Bcl-2 family proteins modulate the mitochondrial apoptotic pathway by interacting with mPTP components, influencing mitochondrial integrity, and governing the release of cytochrome c and other apoptotic factors [62]. Pro-apoptotic proteins such as the multi-BH domain proteins, BAX and BAK as well as the BH3-only proteins, BID and BAD facilitate mitochondrial outer membrane permeabilization (MOMP) and cell death through their interaction with anti-apoptotic proteins like BCl-2 and BCL-XL, whereas anti-apoptotic proteins inhibit apoptosis by sequestering the pro-apoptotic proteins [62, 63]. In addition, the BH3-like domain protein, MOAP-1, interacts with BAX to promote mitochondria dysfunction, cell death and antagonizing the anti-apoptotic activity of BCL-2 and BCL-XL [98]. In leukemia and breast cancer CSCs, overexpression of BCL-2 and BCL-XL has been associated with resistance to chemotherapy, enabling CSCs to evade apoptosis and sustain tumor progression [63, 64].

Targeting Bcl-2 family proteins has emerged as a promising therapeutic strategy to induce apoptosis in CSCs. Small-molecule inhibitors such as ABT-199 (Venetoclax), ABT-737 and Gossypol (AT-101) have demonstrated efficacy in disrupting interactions between BCL-2/BCL-XL and their pro-apoptotic counterparts, thereby restoring apoptotic signaling [65, 99]. For instance, a study demonstrated that ABT-737 effectively induced apoptosis in leukemia stem cells by enhancing BAK/BAX activity and promoting cytochrome c release [65]. Similarly, Parthenolide, a sesquiterpene lactone, has been shown to activate BAX, leading to increased apoptosis in acute myelogenous leukemia, breast cancer, melanoma-initiating cells, and osteosarcoma CSCs [66–68]. Furthermore, Quinacrine, an antimalarial drug repurposed for cancer therapy, has been reported to modulate Bcl-2 family protein activity, triggering apoptosis in CSCs from various tumor types [69–71]. These findings underscore the importance of targeting Bcl-2 family proteins as a therapeutic approach to eliminate CSCs, overcome drug resistance, and improve treatment outcomes in cancer.

### Mitochondrial dysfunction as a therapeutic strategy in CSCs

Mitochondrial dysfunction is a crucial factor in regulating CSC survival and proliferation. Internal and external stimuli can impair mitochondrial function, leading to decreased efficiency of the Electron Transport Chain (ETC), reduced production of high-energy molecules, and subsequent cell death. Since CSCs heavily rely on mitochondrial function to sustain their self-renewal, metabolism, and resistance to therapy, inducing mitochondrial dysfunction presents a promising strategy for CSC-targeted therapies. Various anti-cancer agents, including ceramide, CD437, and MKT077, have been identified as mitochondrial disruptors, selectively inducing apoptosis in CSCs by impairing mitochondrial membrane potential (Δψm) and OXPHOS [72–74].

Clinically approved chemotherapeutic agents such as etoposide, paclitaxel, and vinorelbine have also demonstrated the ability to induce mitochondrial dysfunction [75]. These drugs interfere with mitochondrial bioenergetics and redox homeostasis, sensitizing CSCs to apoptosis [8]. Moreover, targeting metabolic regulators can further disrupt mitochondrial function in CSCs. Fructose-1,6-bisphosphatase (FBP1) downregulation has been linked to enhanced glycolysis and suppression of mitochondrial Complex I activity, driving metabolic reprogramming in CSCs [76]. Metformin, an AMPK activator and Complex I inhibitor, has been extensively studied for its ability to suppress CSCs by impairing mitochondrial respiration and forcing a metabolic shift towards glycolysis [77].

Another emerging strategy involves the use of mitochondrial-targeted agents such as Vitamin E succinate (MitoVES) and Isoflavone derivative NV-128, both of which trigger apoptosis by reducing ATP production and inhibiting mitochondrial Complex I and IV activity [78, 79]. NV-128, in particular, has been shown to significantly decrease ATP levels and mitochondrial respiration in ovarian CSCs, leading to loss of stemness and increased sensitivity to chemotherapy [80]. Additionally, Taxoid SB-T-1214, a next-generation taxoid derivative, has demonstrated efficacy in reducing the expression of key CSC stemness genes, thereby inhibiting their self-renewal potential [81].

Mitochondrial dysfunction can also be leveraged through EpCAM inhibition, which reduces CSC proliferation and differentiation. EpCAM-targeting agents have shown promise in colorectal and ovarian CSCs, significantly impairing their mitochondrial bioenergetics and inhibiting tumor progression [82, 83]. These findings underscore the potential of mitochondrial-targeting drugs to selectively eliminate CSCs by disrupting their metabolic plasticity, reducing stemness, and sensitizing them to conventional therapies. Further research and clinical trials are warranted to optimize mitochondrial-targeted interventions for CSC eradication.

### Tumor necrosis factor receptor (TNFR) signaling in CSCs

The TNFR family plays a critical role in regulating CSC fate by modulating cell death, survival, differentiation, and proliferation. TNF exerts its effects through two primary receptors, TNFR1 and TNFR2, which trigger distinct signaling pathways. TNFR1 activation is generally associated with apoptosis and necroptosis, while TNFR2 primarily promotes survival and proliferation through STAT3 and NF-κB signaling [100]. CSCs exploit TNFR signaling to maintain their stemness and evade apoptosis, making TNFR modulation a potential therapeutic target [101].

A promising strategy to eliminate CSCs involves downregulating TNFR2/STAT3 signaling, which is crucial for CSC maintenance. TNFR2 is known to interact with STAT3, a transcription factor that regulates CSC proliferation and survival. In renal CSCs, the inhibition of TNFR2/STAT3 signaling has been shown to impair mitochondrial function by altering mitochondrial ultrastructure, increasing cytochrome c translocation to the cytoplasm, and activating cleaved caspase-3 p175, leading to apoptotic cell death [84].

Furthermore, TNF signaling influences CSC metabolism by targeting ETC Complex I and II, thereby disrupting ATP production and mitochondrial homeostasis. The dephosphorylation of STAT3 on serine-727 (pSTAT3Ser727) has been linked to TNF-mediated cell death, reducing the ability of CSCs to sustain OXPHOS and survive under metabolic stress [85, 86]. These findings suggest that targeting TNFR signaling in CSCs, particularly through TNFR2 downregulation and STAT3 inhibition, can induce apoptosis, disrupt mitochondrial metabolism, and impair CSC survival. Future research should explore the combination of TNFR modulators with conventional chemotherapies to enhance CSC eradication and improve clinical outcomes.

## Conclusion

Mitochondria play a central role in regulating CSC survival, metabolic adaptation, and therapeutic resistance, making them a crucial target for innovative cancer treatments. The intricate interplay between mitochondrial dynamics, signaling pathways, and stress responses enables CSCs to evade apoptosis, sustain energy production, and drive tumor progression. By modulating key mitochondrial processes such as mitophagy, biogenesis, OXPHOS, and ROX regulation, CSCs maintain their plasticity and resilience against conventional therapies. Targeting mitochondrial vulnerabilities, including dysregulated fission-fusion balance, membrane permeability transition, and apoptotic resistance, offers promising therapeutic strategies to eliminate CSCs and prevent tumor recurrence. Future research should focus on developing precise mitochondrial-targeted therapies that disrupt CSC metabolism while minimizing toxicity to normal stem cells. Understanding the molecular intricacies of mitochondrial function in CSCs will pave the way for novel interventions that enhance treatment efficacy and improve clinical outcomes in cancer patients.

## Data Availability

No datasets were generated or analysed during the current study.

## Abbreviations

Δψm: Mitochondrial membrane potential
2-HG: 2-hydroxyglutarate
5-FU: 5-fluorouracil
ABC: ATP binding cassette
AMPK: AMP-activated protein kinase
ANT: Adenine nucleotide translocator
ATF4: Activating transcription factor 4
ATP: Adenosine Triphosphate
Bcl-2: B-cell lymphoma protein 2
BCL-XL: B-cell lymphoma-extra large
BNIP3: BCL2 interacting protein 3
Ca²⁺: Calcium ion
CD: Cluster of differentiation
CHOP: C/EBP Homologous Protein
CSC: Cancer stem cell
CypD: Cyclophilin D
DRP1: Dynamin-related protein 1
eIF2α: Eukaryotic initiation factor 2
EpCAM: Epithelial cellular adhesion molecule
ERRα: Estrogen receptor related receptor
ETC: Electron transport chain
FBP1: Fructose-1,6-bisphosphatase
FOXM1: Forkhead box M1
FUNDC1: FUN14 domain-containing protein 1
GTP: Guanosine Triphosphate
HIF-1α: Hypoxia-Inducible Factor-1 Alpha
ISR: Integrated stress response
KRAS: Kirsten rat sarcoma viral oncogene homolog
MAPK: Mitogen-activated protein kinase
MitoVES: Vitamin E succinate
MOMP: Mitochondrial outer membrane permeabilization
MPT: Membrane permeability transition
mPTP: Mitochondrial Permeability Transition Pore
mtDNA: Mitochondrial DNA
mTOR: Mammalian target of rapamycin
NIX: NIP3-like protein X
NRF: Nuclear respiratory factor
OXPHOS: Oxidative phosphorylation
PERK: PKR-like endoplasmic reticulum kinase
PGC-1α: Peroxisome proliferator-activated receptor-gamma coactivator
PINK1: PTEN-induced kinase 1
PRDX3: Peroxiredoxin 3
ROS: Reactive Oxygen Species
SIRT: Silent information regulator sirtuin
STAT3: Signal transducer and activator of transcription 3
TFAM: Mitochondrial transcription factor A
TNFR: Tumor necrosis factor receptor
TSPO: Translocator protein
ULK1: Autophagy activating kinase 1
UPR^mt^: Mitochondrial unfolded protein response
VDAC1: Voltage-dependent anion channel 1
